# Genome-wide characterization of *AINTEGUMENTA-LIKE* family in *Medicago truncatula* reveals the significant roles of *AINTEGUMENTAs* in leaf growth

**DOI:** 10.3389/fpls.2022.1050462

**Published:** 2022-11-03

**Authors:** Xiao Wang, Juanjuan Zhang, Jing Zhang, Chuanen Zhou, Lu Han

**Affiliations:** The Key Laboratory of Plant Development and Environmental Adaptation Biology, Ministry of Education, School of Life Sciences, Shandong University, Qingdao, China

**Keywords:** *Medicago truncatula*, *AILs*, *ANT*, leaf size, leaf morphogenesis

## Abstract

*AINTEGUMENTA-LIKE* (AIL) transcription factors are widely studied and play crucial roles in plant growth and development. However, the functions of the *AIL* family in legume species are largely unknown. In this study, 11 *MtAIL* genes were identified in the model legume *Medicago truncatula*, of which four of them are *MtANTs*. *In situ* analysis showed that *MtANT1* was highly expressed in the shoot apical meristem (SAM) and leaf primordium. Characterization of *mtant1 mtant2 mtant3 mtant4* quadruple mutants and *MtANT1*-overexpressing plants revealed that *MtANTs* were not only necessary but also sufficient for the regulation of leaf size, and indicated that they mainly function in the regulation of cell proliferation during secondary morphogenesis of leaves in *M. truncatula*. This study systematically analyzed the *MtAIL* family at the genome-wide level and revealed the functions of *MtANTs* in leaf growth. Thus, these genes may provide a potential application for promoting the biomass of legume forages.

## Introduction

The AINTEGUMENTA-LIKE (AIL) transcription factors have been widely studied in plants (Klucher et al., 1996; Mizumoto et al., 2009; Karlberg et al., 2011; Li and Xue, 2011; Rigal et al., 2012; Horstman et al., 2014; Kuluev et al., 2015; Bui et al., 2017; Ding et al., 2018; Zhao et al., 2019b; Liu et al., 2020; Shen et al., 2020; Miao et al., 2021; Han et al., 2022). They belong to the APETALA 2 (AP2)-like subfamily, which is characterized by two putative DNA-binding AP2 domains and one conserved linker region (Kim et al., 2006). The AP2-like subfamily can be divided into three groups: euAP2, basalANT, and euANT (Kim et al., 2006). Unlike *euAP2* genes, the *basalANT* and *euANT* genes can not be recognized by *miR172* and are differentiated by specific sequence signatures (Kim et al., 2006; Dipp-Álvarez and Cruz-Ramírez, 2019).

In *Arabidopsis thaliana*, the euANT proteins are also known as AILs and consist of *ANT*, *AIL1*, *AIL2*/*BBM*/*PLT4*, *AIL3*/*PLT1*, *AIL4*/*PLT2*, *AIL5*/*PLT5*, *AIL6*/*PLT3* and *AIL7*/*PLT7* (Nole-Wilson et al., 2005; Horstman et al., 2014). *ANT* mainly regulates cell division and cell differentiation in leaves and floral organs. For instance, *ANT* regulates the size of lateral organs by controlling cell proliferation during organogenesis (Mizukami and Fischer, 2000). *ANT* also participates in the establishment of adaxial-abaxial polarity. *ANT* acts with the abaxial-specifying gene *FILAMENTOUS FLOWER* (*FIL*) to up-regulate the expression of the adaxial gene *PHABULOSA* (Nole-Wilson and Krizek, 2006). Meanwhile, *ANT* and *AIL5*/*6*/*7* function partially redundantly in flower development, and the direct targets of *ANT* and *AIL6* include *LEAFY* and other genes involved in polarity establishment, meristem and flower development, as well as auxin signaling pathway (Krizek, 2009; Krizek, 2015; Yamaguchi et al., 2016; Krizek et al., 2020; Krizek et al., 2021). *ANT*, *AIL6*, and *AIL7* regulate SAM function; the SAM of the *ant ail6 ail7* triple mutant terminates after the production of only a few leaves (Mudunkothge and Krizek, 2012). Furthermore, *BBM*/*AIL2*, *PLT1*/*AIL3*, *PLT2*/*AIL4* and *PLT3*/*AIL6* regulate cell proliferation during embryogenesis and root apical meristem maintenance (Horstman et al., 2014). These genes function in stem cell identity in the root and promote cell division of the stem cell daughters (Galinha et al., 2007). *PLT1*/*AIL3* and *PLT2*/*AIL4* also play essential roles in the quiescent center specification and stem cells maintenance (Aida et al., 2004). *BBM*/*AIL2* is involved in the embryo and endosperm development, and ectopic expression of *BBM*/*AIL2* leads to the production of somatic embryos on seedlings (Boutilier et al., 2002; Chen et al., 2022). Moreover, *AIL5*, *AIL6*, and *AIL7* execute an extra function in phyllotaxy stability and lateral root emergence (Prasad et al., 2011; Hofhuis et al., 2013; Pinon et al., 2013).

The roles of *AIL* genes in panicle, root, seed, leaf, flower, and chloroplast development have been reported in other species. *OsAILs* are involved in panicle branching, panicle structure regulation, and crown root initiation in rice (Kitomi et al., 2011; Harrop et al., 2019; Luong et al., 2021). In poplar, *PtAIL1* plays a positive role in adventitious root formation (Rigal et al., 2012). In *Medicago truncatula*, ectopic expression of *AtANT* under the control of a seed-specific promoter generates larger seeds and improves the germination rate (Confalonieri et al., 2014). In maize, *ZmANT1* regulates leaf and vascular tissue development, chloroplast development, and photosynthesis (Liu et al., 2020). In *Nicotiana tabacum*, *Cucurbita moschata*, *Triticum aestivum*, and *Brassica rapa*, putative orthologs of *ANT* positively regulate organ size. For example, *NtANT* increases the size of leaf and corolla by promoting cell division and expansion (Kuluev et al., 2015), ectopic expression of *CmoANT* accelerates the growth of grafted plants and promotes the size of silique and leaf (Miao et al., 2021), ectopic expression of *TaANT* enlarges plant size by promoting cell proliferation (Zhao et al., 2019b), and ectopic expression of the *BrANT* increases stomatal density and organ size (Ding et al., 2018).

Alfalfa (*Medicago sativa*) has the characteristics of high biomass yield, good forage quality, high adaptability to growing conditions, and palatability for ruminants, and has been called the “Queen of Forage” (Radović et al., 2009). Alfalfa cultivars are allogamous, self-incompatibly, autotetraploid plants with a complex genome, which leads to difficulty in genomics research (Zhu et al., 2005). *Medicago truncatula* has been adopted as a model legume species for a range of genetics and genomics studies. The *AIL* genes have been well studied in many species, but the information and functions of *AIL* genes are largely unknown in legume species. In this study, the genome-wide identification and characterization of the *AIL* gene family was performed in *M. truncatula*. Eleven *MtAIL* genes were identified and their phylogenetic relationship, gene structure, and protein motifs were analyzed. Furthermore, the expression patterns of *MtANTs* showed that *MtANT1* was highly expressed in the SAM and leaf primordium. Loss-of-function mutants of *MtANTs* were isolated and the *mtant1 mtant2 mtant3 mtant4* quadruple mutant was generated. The quadruple mutant exhibited obvious defects in leaf size, while, transgenics overexpressing *MtANT1* produced enlarged leaves. Cellular level analysis indicates that *MtANTs* regulate leaf size mainly through cell proliferation. Our study provides detailed information on the *MtAILs* and demonstrates that *MtANT* genes play vital roles in leaf growth in *M. truncatula*.

## Materials and methods

### Plant material and growth conditions

*Medicago truncatula* ecotype R108 was used as the wild type in this study. *mtant1-1*, *mtant2-1*, *mtant3-1*, and *mtant4-1* were identified from a tobacco (*Nicotiana tabacum*) *Tnt1* retrotransposon-tagged mutant population of *M. truncatula* (Tadege et al., 2008). The seeds were scarified with sandpaper and treated at 4°C for 7 days. The germinated seeds were planted in a nursery seedling plate for 3 weeks. Then, the seedlings were transferred to soil and grown at 22°C ± 2°C under long-day conditions (16-h light and 8-h dark), with a relative humidity of 70%–80%.

### Identification and phylogenetic analysis of AIL genes in *M. truncatula*

To identify the AIL proteins in *M. truncatula*, 8 AILs in *Arabidopsis thaliana* and 10 AILs in *Oryza sativa* were used to execute BLASTP Search against the sequence database of the *Medicago truncatula* in Phytozome (https://phytozome-next.jgi.doe.gov/). We selected the fullest transcripts for the study, and other splice variants were excluded. To investigate the phylogenetic relationships of AILs in different species, 8 AIL proteins in *Arabidopsis thaliana*, 10 AIL proteins in *Oryza sativa*, 11 AIL proteins in *Pisum sativum*, 9 AIL proteins in *Lotus japonicus*, 18 AIL proteins in *Glycine max* and 11 identified AIL proteins in *Medicago truncatula* were used to construct the phylogenetic tree. The phylogenetic trees were generated with the Neighbor-Joining method and 1000 Bootstrap Replications using MEGA6.06 (Kumar et al., 2008). The amino acid sequences of the AtAILs were obtained from The Arabidopsis Information Resource (TAIR) database (http://www.Arabidopsis.org/). The OsAILs protein sequences were obtained from the Rice Genome Annotation Project (http://rice.plantbiology.msu.edu/) and National Center for Biotechnology Information (https://www.ncbi.nlm.nih.gov/ ). The GmAILs and LjAILs protein sequences were obtained in Phytozome, and the PsAILs protein sequences were obtained in the Pea Genome Database (https://www.peagdb.com/index/ ) (Yang et al., 2022).

### Gene structure, conserved domains and motif analysis

Exon and intron structures analysis of MtAIL genes were determined by aligning the CDS sequences and their corresponding genomic DNA sequences using the Gene Structure Display Server (GSDS 2.0, http://gsds.gao-lab.org/ ) (Wang et al., 2019). Conserved motifs in MtAIL proteins were analyzed with the Multiple Em for Motif Elicitation (MEME, https://meme-suite.org/meme/tools/meme ) with the following parameters: maximum of motif width, 80; minimum width of motif, 4; maximum motif number, 10 (Wang et al., 2018). The MtAIL proteins were aligned using Clustal X2 (Larkin et al., 2007), and GeneDoc software was used for homology shading (Brocard-Gifford et al., 2004).

### RT-PCR and qRT-PCR analysis and statistical analysis

For gene expression pattern analysis, total RNA was extracted from the leaves, vegetative buds, flowers, stems, petioles, pods, and roots. To analyze the relative expression levels of *MtANT1* in the overexpressing plants, RNA was extracted from 30-d-old mature leaves of wild type and transgenic plants. For RT-PCR analysis, RNA was extracted from vegetative buds of wild type and mutant lines. RNA extraction, cDNA synthesis, qRT-PCR, and RT-PCR analyses were performed as described previously (Zhou et al., 2011). The primers used for qRT-PCR and RT-PCR are listed in **Supplementary Table S1**. *T*-test was used to compare the means of different populations.

### *In situ* hybridization analysis

For RNA *in situ* hybridization, the 583-bp CDS of *MtANT1*, the 546-bp CDS of *MtANT2*, and the 502-bp CDS of *MtANT3* were amplified. The PCR products were cloned into the pGEM-T vector (Promega). The sense and anti-sense probes were made according to the previous report (Zhao et al., 2019a). 30-d-old wild type vegetative buds were used for RNA *in situ* hybridization as previously described (Zhou et al., 2011). The primers used for RNA *in situ* hybridization are listed in **Supplementary Table S1**

### Plasmids and plant transformation

To obtain the *MtANT1* overexpression construction, the full-length CDS of *MtANT1* was obtained by PCR amplification and inserted into the pENTR/D-TOPO vector (Invitrogen), and then recombined with final vector pEarleyGate 100, using the Gateway LR reactions (Invitrogen) (Earley et al., 2006). The primers used are listed in **Supplementary Table S1**. The *35S:MtANT1* construct was introduced into *Agrobacterium* strain EHA105. For stable transformation, leaves of wild type were used for transformation (Cosson et al., 2006).

### SEM analysis

SEM was performed as described previously (Zhang et al., 2022). Briefly, leaves were fixed, dehydrated, critical point dried, and observed for imaging.

## Results

### Identification and phylogenetic analysis of AILs in *M. truncatula*

**Figure 1:**
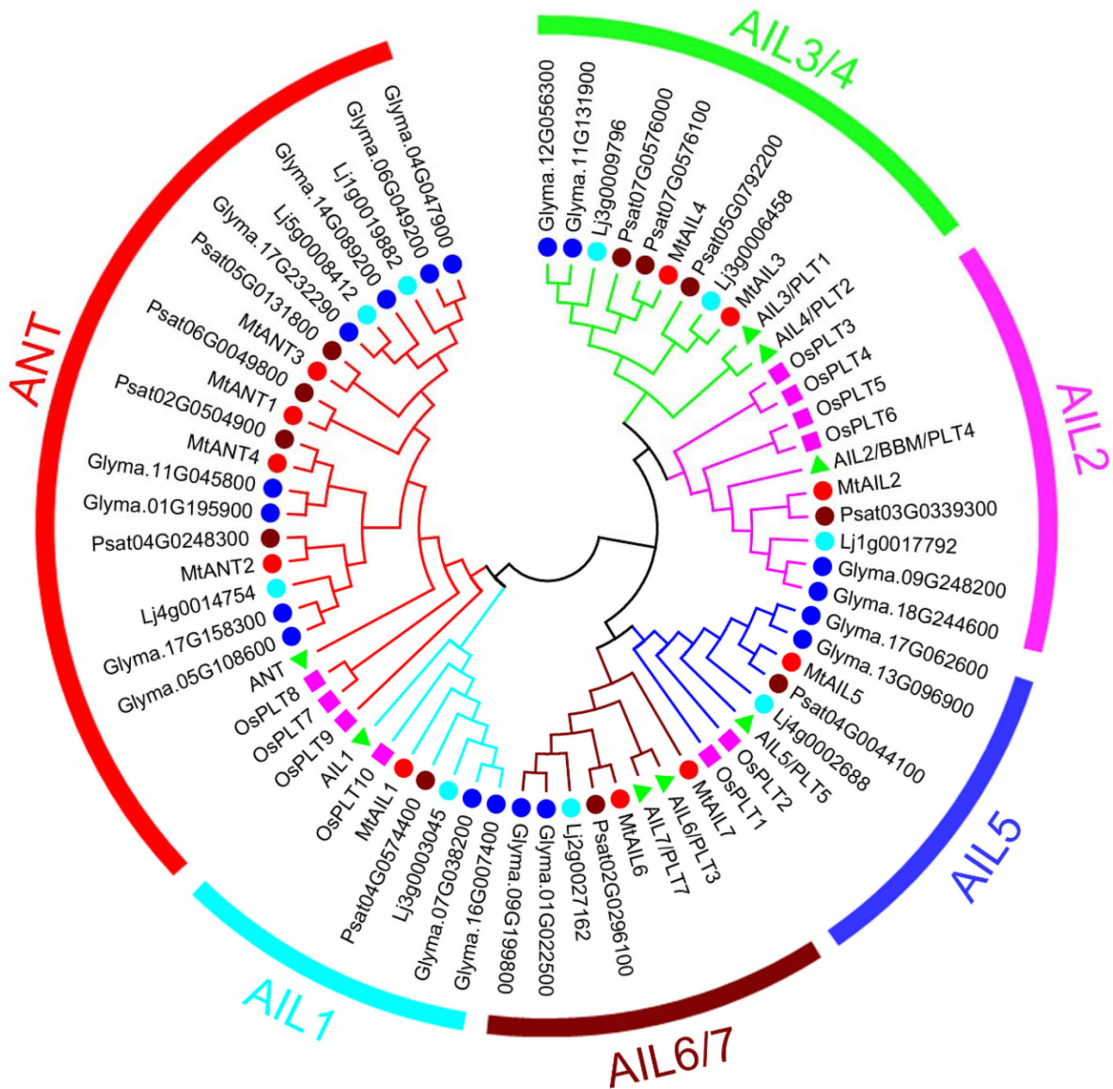
Phylogenetic tree analysis of AIL transcription factors from *Medicago truncatula*, *Arabidopsis thaliana*, *Oryza sativa*, *Pisum sativum*, *Lotus japonicus* and *Glycine max*. The Neighbor-Joining tree was constructed using full length protein sequences from *M. truncatula* (11), *Arabidopsis* (8), *Oryza sativa* (10), *Pisum sativum* (11), *Lotus japonicus* (9) and *Glycine max* (18) in MEGA6.06 with 1000 bootstrap replicates. The red, brown, light blue and blue circles indicate *M. truncatula*, *Pisum sativum*, *Lotus japonicus* and *Glycine max*, respectively. The pink boxes and green triangles indicate *Oryza sativa* and *Arabidopsis*.

Eleven putative MtAIL proteins were identified by BLAST Search in Phytozome. The length of 11 MtAILs proteins ranged from 402 to 688 amino acids. The gene locus, exon number, amino acid length, molecular weight (Mw), and chromosome location are listed in **Table 1**. Based on the gene locus, these *MtAIL* genes showed uneven distribution on the *M. truncatula* chromosomes. Chromosome 1, 2, 3, and 7 contained one *MtAIL* gene, respectively. Both chromosome 5 and 8 contained two *MtAIL* genes, chromosome 4 contained four *MtAIL* genes, and no *MtAIL* gene was located on chromosome 6 (**Table 1**). To further investigate the evolutionary relationship between MtAIL proteins and homologs in other species, a phylogenetic tree was constructed, including 8 AtAILs, 10 OsAILs, 9 LjAILs, 18 GmAILs, 11 PsAILs and 11 MtAILs (**Figure 1**). Based on the phylogenetic analysis, all the *MtAIL* genes were named according to their closest *Arabidopsis* orthologs. Furthermore, 67 AIL proteins were classified into six clades: ANT, AIL1, AIL2, AIL3/AIL4, AIL5, and AIL6/7. MtAILs were close to PsAILs, and GmAILs were more closely related to LjAILs. Moreover, every GmAIL gene contained more than one copy. OsAILs were separated from others mainly because of the species differences. ANT clade contained four members of MtAILs which were named MtANT1 to MtANT4. Clades AIL1, AIL2, AIL3/AIL4, AIL5, and AIL6/7 contained seven MtAILs which were named MtAIL1 to MtAIL7 (**Figure 1**). Phylogenetic analysis also showed that MtAIL1 was more closely related to MtANTs, MtAIL2 was close to MtAIL3 and MtAIL4, MtAIL5 was clustered with MtAIL6 and MtAIL7 (**Figure 1**).

**Table 1:** *AIL* gene family in *M. truncatula* .

Name	Locus	CDS (nt)	Exons	Length (aa)	MW (kDa)	Chromosome location
*MtANT2*	Medtr4g097520	1977	9	658	73.8	chr4:40188318..40192060 forward
*MtANT1*	Medtr1g017400	1995	9	664	74.13	chr1:4844539..4848969 reverse
*MtANT3*	Medtr3g103460	1986	9	661	73.18	chr3:47751101..47755318 forward
*MtAIL1*	Medtr8g020510	1722	9	573	63.95	chr8:7209111..7212535 forward
*MtANT4*	Medtr5g015070	1635	9	544	61.52	chr5:5176272..5179958 reverse
*MtAIL5*	Medtr4g127930	1557	9	518	55.82	chr4:53232819..53237003 reverse
*MtAIL2*	Medtr7g080460	2067	9	688	76.57	chr7:30617122..30621534 reverse
*MtAIL3*	Medtr2g098180	1578	9	525	58.7	chr2:41962850..41966348 reverse
*MtAIL4*	Medtr4g065370	1644	9	547	61.25	chr4:24560916..24564307 reverse
*MtAIL6*	Medtr5g031880	1545	9	514	56.95	chr5:13680654..13684967 reverse
*MtAIL7*	Medtr8g068510	1209	9	402	44.79	chr8:28586113..28591359 reverse

### Gene structures and conserved motifs analysis

**Figure 2:**
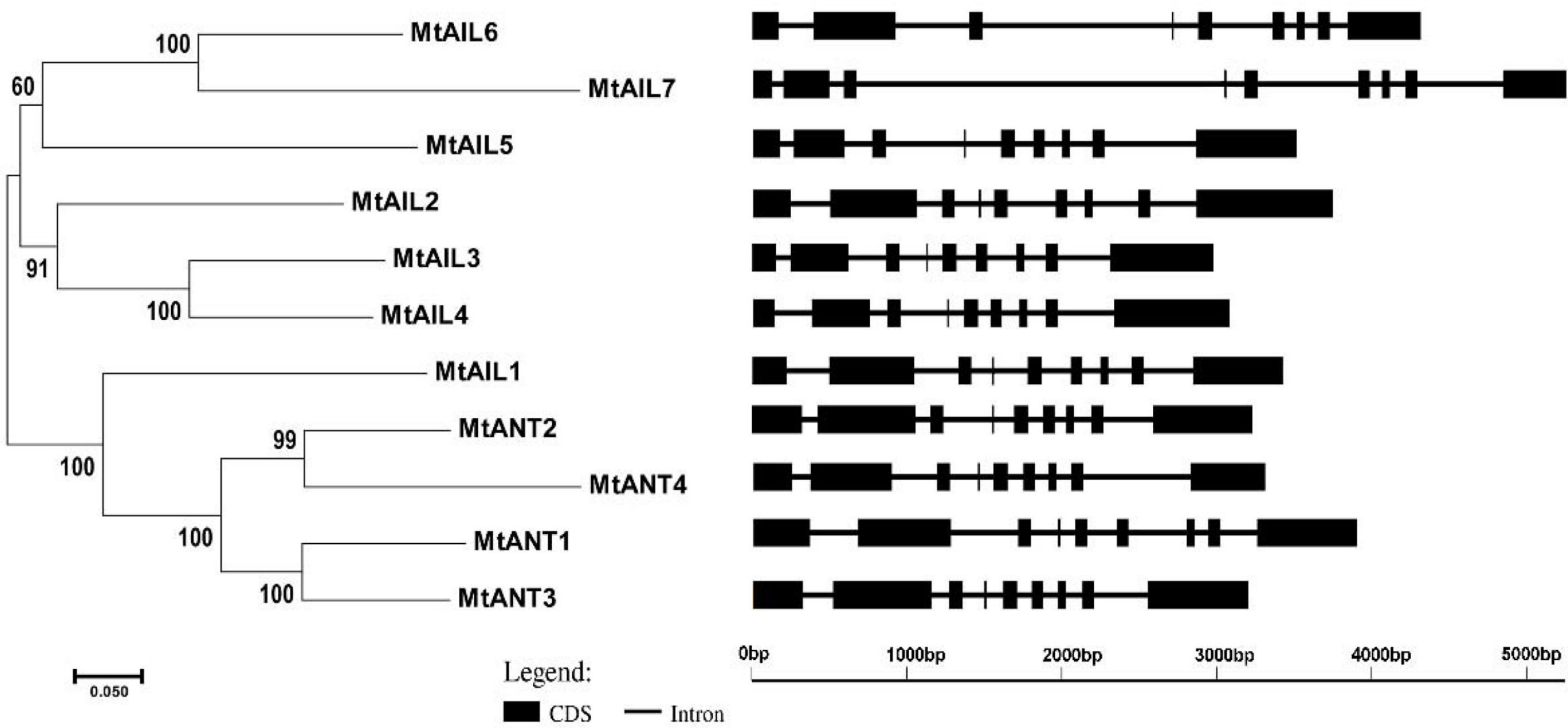
The phylogenetic relationship and exon/intron structural analyses of all *AILs* in *M. truncatula*. Introns are represented by black lines and exons are represented by black boxes. Phylogenetic tree was constructed using MEGA6.06 by the Neighbor-Joining method.

**Figure 3:**
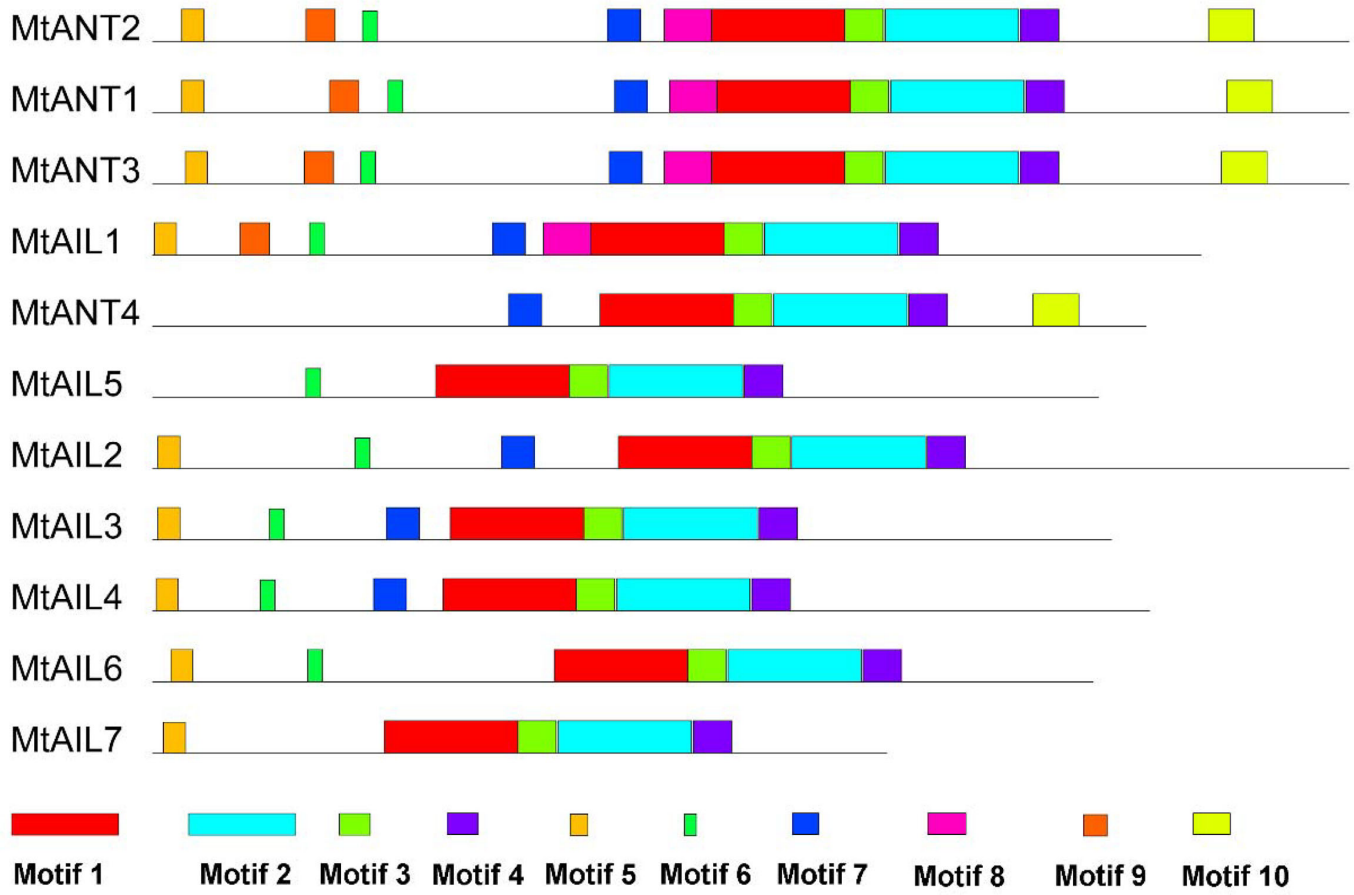
Motifs in MtAIL proteins. The motifs in MtAILs were indicated using MEME online tool. Motifs were represented by the boxes with different numbers and colors.

To further study the diversification of *MtAILs*, the corresponding gene structures of *MtAILs* were analyzed. The exon/intron organization of *MtAILs* was investigated by aligning the coding sequences and corresponding genomic sequences. All the *MtAILs* displayed 9 exons and 8 introns (**Figure 2**), suggesting that the gene structures of the *MtAIL* family are conserved during evolution. Previous studies showed that AILs belong to the AP2-like transcription factor subfamily, which is characterized by two AP2 domains (Nole-Wilson et al., 2005; Licausi et al., 2013). According to this, the amino acid sequences of MtAILs were aligned, and two conserved AP2 domains were shown in MtAILs, including the N-terminal AP2-R1 and C-terminal AP2-R2 domains (**Supplementary Figure S1**). To get a better understanding of the protein sequence characteristics of MtAILs, the motifs of MtAILs were analyzed. Online MEME search was performed and 10 conserved motifs were identified in MtAILs (**Figure 3**). The motifs 1, 2, 3, 4 were found in each MtAIL. Among them, motifs 1, 2 and 3 were found to be similar to the AP2-R1-linker-AP2-R2 region of AP2-like proteins. Motif 4 was located downstream of the AP2-R2 domain and sequence analysis suggests that it may function as a nuclear localization signal (Aida et al., 2004; Dipp-Álvarez and Cruz-Ramírez, 2019). Motif 5 contained the euANT2 motif (WLGFSLF), and motif 6 was the euANT3 motif (PKLEDFLG). Motif 5 and 6 were conserved motifs of euANT clade protein and existed in almost all the MtAILs (Kim et al., 2006; Dipp-Álvarez and Cruz-Ramírez, 2019). Motif 7 was absent in MtAIL5, 6, 7 proteins, suggesting that motif 7 is lost before the MtAIL5, 6, 7 divergence. Motifs 8 and 9 were specific to MtANT1, 2, 3 and MtAIL1, while motif 10 was only presented in MtANTs, implying that the distribution of motifs among specific groups is related to their functional divergence (**Figure 3**).

### Expression patterns of *MtANTs* in *M. truncatula*

**Figure 4:**
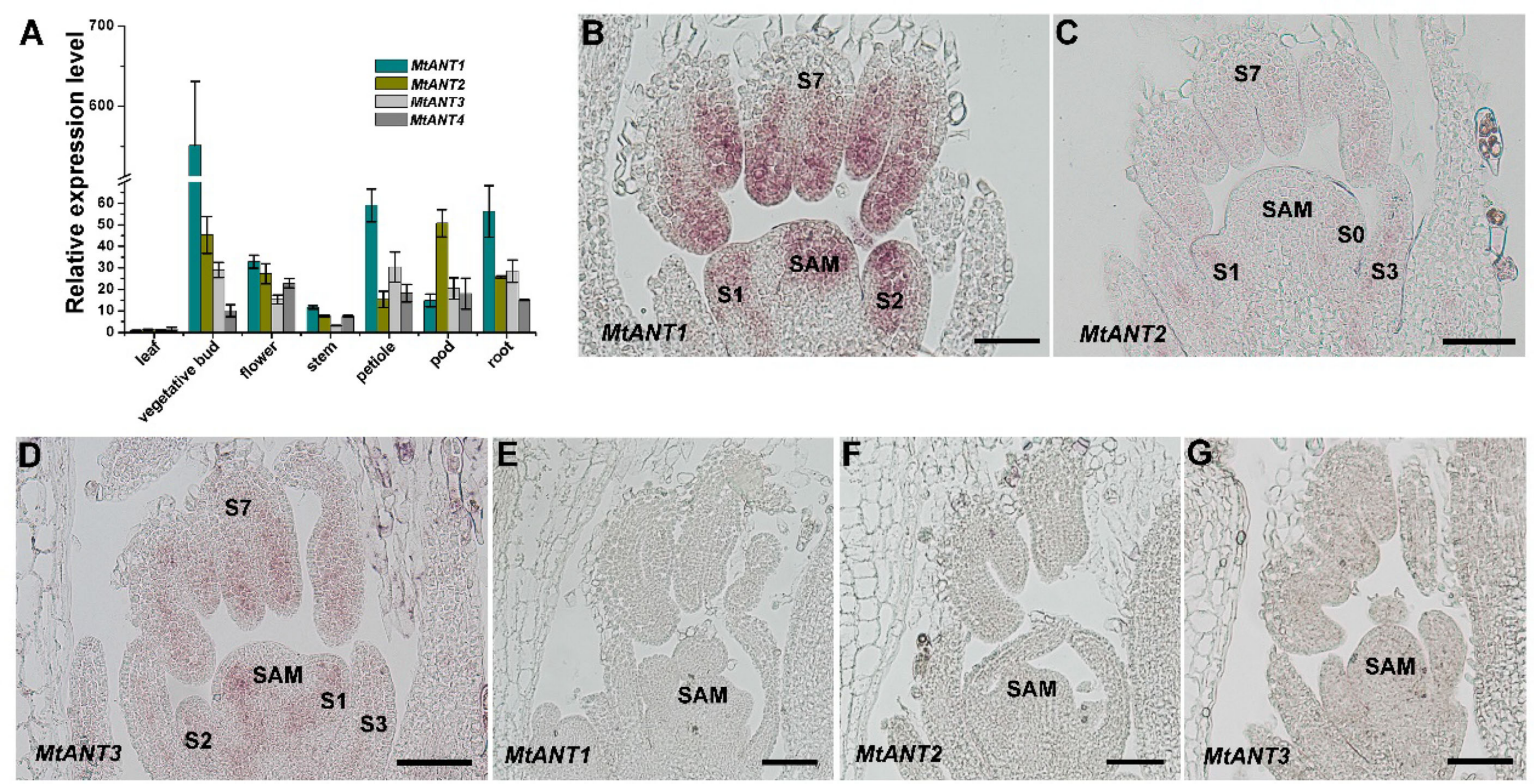
Expression patterns of *MtANTs* in *M. truncatula*. **(A)** qRT-PCR analysis of *MtANTs* expression in different organs. *MtUBIQUITIN* was used as the internal control. Bars represent means ± SD of three biological replicates. **(B-D)** *In situ* hybridization analysis of *MtANT1* **(B)**, *MtANT2* **(C)** and *MtANT3* **(D)** in the vegetative buds of wild type. Longitudinal sections of SAM and leaf primordia at the different developmental stages are shown. SAM, shoot apical meristem; S, stage. Bars = 100 μm. **(E–G)** The sense probes were used as the negative controls. SAM, shoot apical meristem; Bars = 100 μm.

In *Arabidopsis*, *ANT* plays an important role in the regulation of organ initiation, organ development, and cell proliferation (Mizukami and Fischer, 2000; Horstman et al., 2014). In order to investigate the function of *MtANTs*, the expression patterns of *MtANTs* were analyzed. The expression levels of *MtANT1*, *MtANT2*, *MtANT3*, and *MtANT4* were measured by quantitative real-time PCR (qRT-PCR) in different organs, including leaf, vegetative bud, flower, stem, petiole, pod, and root. qRT-PCR results showed that the relative expression levels and patterns of *MtANTs* varied in different organs, but all the *MtANTs* were expressed at the lowest levels in leaves (**Figure 4A**). Among the four *MtANTs*, the expression of *MtANT1* was much higher than the other three genes in vegetative buds. Additionally, the expression level of *MtANT1* in vegetative buds was higher than other tissues (**Figure 4A**). To gain spatial information about the expression patterns, *in situ* hybridization was performed for *MtANT1*, *MtANT2*, and *MtANT3*. *In situ* hybridization was not performed for *MtANT4* because it showed very low expression levels in vegetative buds. Strong *MtANT1* signals were detected in SAM, leaf primordia at stage 1 and stage 2, and leaves at stage 7 (**Figure 4B**). *MtANT2* and *MtANT3* transcripts were not detected in the SAM, and fewer transcripts were detected in leaf primordia and leaves (**Figures 4C–D**). The overall transcript level of *MtANT1* was much higher than those of *MtANT2* and *MtANT3* (**Figures 4B–D**). The sense probes were used as the negative controls and did not show any signal (**Figures 4E–G**).

### *MtANTs* are necessary for leaf size maintenance

**Figure 5:**
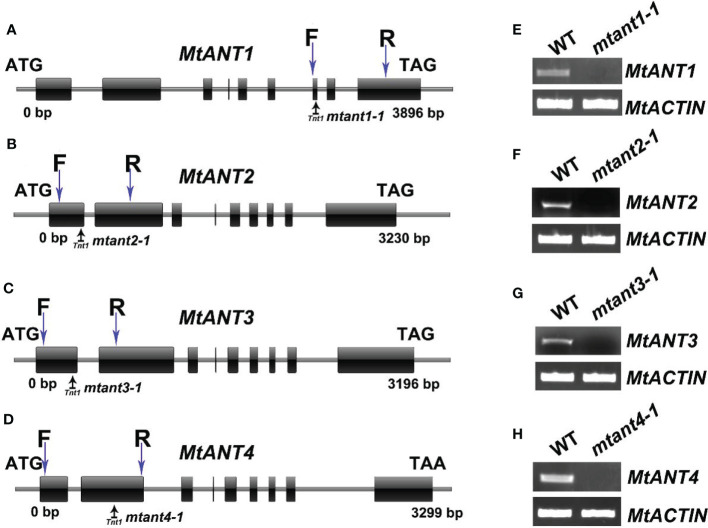
Mutant screening of *MtANTs* in *M. truncatula*. **(A–D)** Schematic diagram of the gene structures of *MtANT1*-*MtANT4*. The positions of the ATG start and TGA/TAA stop codons are shown. Black vertical arrows mark the location of *Tnt1* retrotransposons. Blue vertical arrows mark the location of primers used for RT-PCR. Exon is represented by a box, and intron is represented by a line. **(E–H)** RT-PCR shows the transcripts of *MtANTs* in wild type and *mtant* mutants. *MtACTIN* was used as the control.

**Figure 6:**
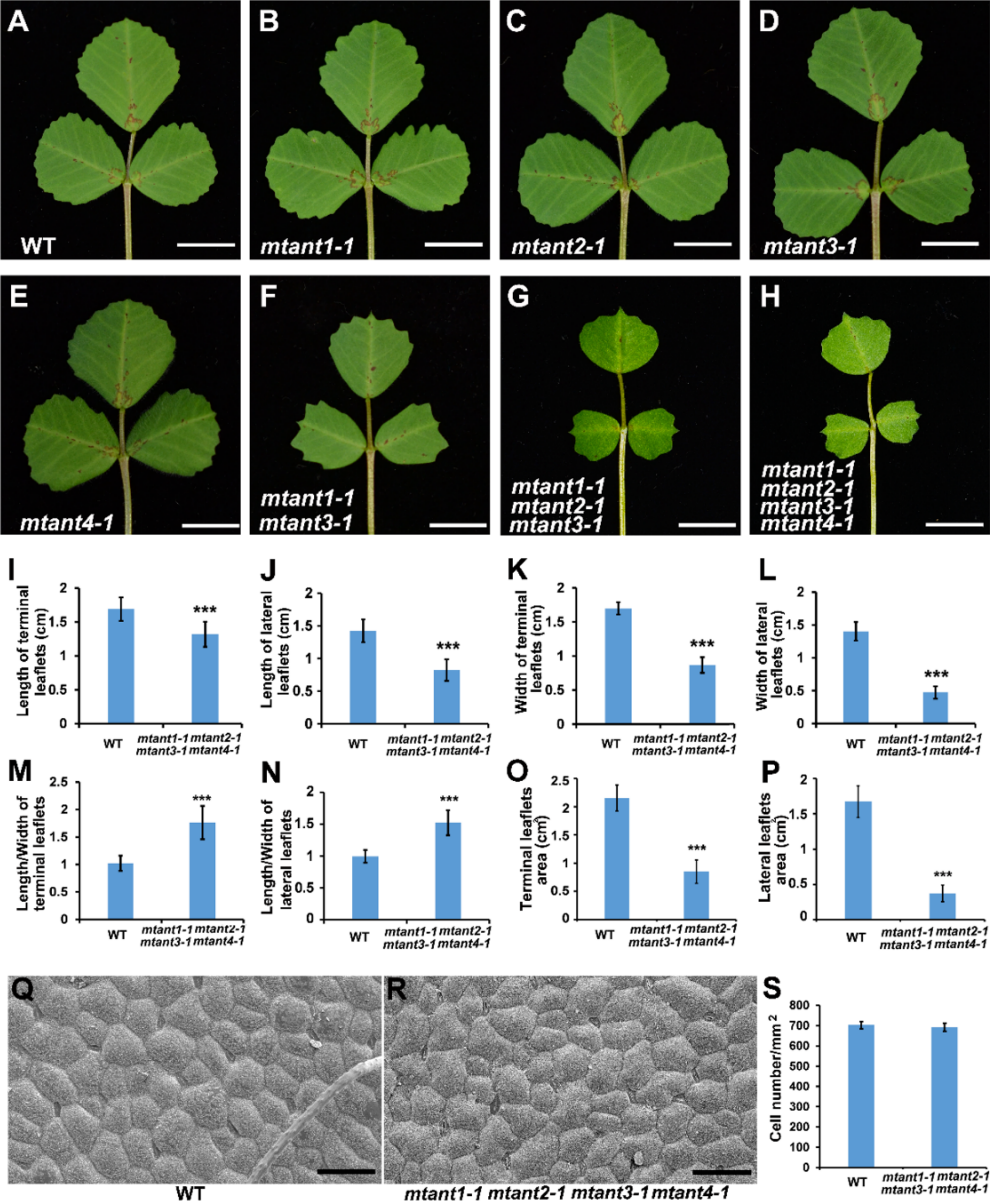
Phenotype analyses of leaves in mtant mutants. **(A–H)** Leaf phenotypes of wild type **(A)**, mtant1-1 **(B)**, mtant2-1 **(C)**, mtant3-1 **(D)**, mtant4-1 **(E)**, mtant1-1 mtant3-1 **(F)**, mtant1-1 mtant2-1 mtant3-1 **(G)** and mtant1-1 mtant2-1 mtant3-1 mtant4-1 **(H)**. Bars = 1 cm. **(I–L)** Leaf length **(I–J)** and width **(K, L)** in terminal leaflets and lateral leaflets of wild type and mtant1-1 mtant2-1 mtant3-1 mtant4-1. **(M–P)** Length/width ratio **(M, N)** and leaf area **(O, P)** of terminal leaflets and lateral leaflets in wild type and mtant1-1 mtant2-1 mtant3-1 mtant4-1. **(Q, R)** Scanning electron microscopy analyses of the adaxial surface of leaves in wild type **(Q)** and mtant1-1 mtant2-1 mtant3-1 mtant4-1 mutant **(R)**. Bars = 50 μm. **(S)** Cell number per unit area of the adaxial surface of leaves in the wild type and mtant1-1 mtant2-1 mtant3-1 mtant4-1 mutant. Bars represent means ± SD. ***P < 0.001.

To investigate the function of *MtANTs* in leaf morphogenesis, a reverse genetic screening was performed on a *Tnt1* retrotransposon-tagged mutant population of *M. truncatula* (Cheng et al., 2014). Insertional mutant alleles were identified in *MtANT1*, *MtANT2*, *MtANT3* and *MtANT4*. Sequence analysis showed that a single *Tnt1* was inserted in the sixth exon of *MtANT1* in *mtant1-1*, the first exon of *MtANT2* in *mtant2-1*, the first exon of *MtANT3* in *mtant3-1*, and the second exon of *MtANT4* in *mtant4-1* (**Figures 5A–D**). Reverse transcription PCR (RT-PCR) data showed that the transcripts of *MtANT1-4* were interrupted in the *mtant1-1*, *mtant2-1*, *mtant3-1*, and *mtant4-1* mutants, respectiveley (**Figures 5E–H**). Subsequently, the leaves of mutants were observed. Compared with the wild type, *mtant1-4* mutants did not show obvious defects in leaf morphology and compound leaf pattern (**Figures 6A–E**). To assess functional redundancy among *MtANTs*, *mtant1-1 mtant3-1* and *mtant2-1 mtant4-1* double mutants were generated. The *mtant1-1 mtant3-1* double mutant exhibited relatively smaller leaves (**Figure 6F**), but the *mtant2-1 mtant4-1* double mutant did not show obvious differences in leaf pattern compared with wild type **(** **Supplementary Figure S2**). Then, high order mutants were generated in the *mtant1-1* background, since *MtANT1* showed the strongest expression in leaf primordia. Simultaneous disruption of *MtANT1*, *2*, *3* or *MtANT1*, *2*, *3*, *4* resulted in a significantly smaller leaf phenotype than that in the wild type (**Figures 6G, H**). It was worth noting that the leaf phenotypes of *mtant1-1 mtant2-1 mtant3-1* triple mutant and *mtant1-1 mtant2-1 mtant3-1 mtant4-1* quadruple mutant were similar (**Figures 6G, H**), indicating that *MtANT4* play a limited role in leaf development. Moreover, the length and width of leaves in *mtant1-1 mtant2-1 mtant3-1 mtant4-1* were reduced compared with those of wild type (**Figures 6I–L**). Compared with the wild type, the length/width ratio was increased, and the leaf area was significantly decreased in *mtant1-1 mtant2-1 mtant3-1 mtant4-1* (**Figures 6M–P**), demonstrating that simultaneous disruption of *MtANT1*, *2*, *3*, *4* resulted in smaller leaves in *M. truncatula*. To explore the cellular basis for the alteration in leaf dimensions of wild type and *mtant1-1 mtant2-1 mtant3-1 mtant4-1* mutant, we viewed the epidermal cells by scanning electron microscopy (SEM). Results showed that no differences were found for both epidermal cell size and cell number per unit area in the wild type and *mtant* quadruple mutant (**Figures 6Q–S**), indicating that the smaller leaf area of *mtant* quadruple mutant was resulted from cell proliferation.

### *MtANT1* is sufficient for increasing leaf size

**Figure 7:**
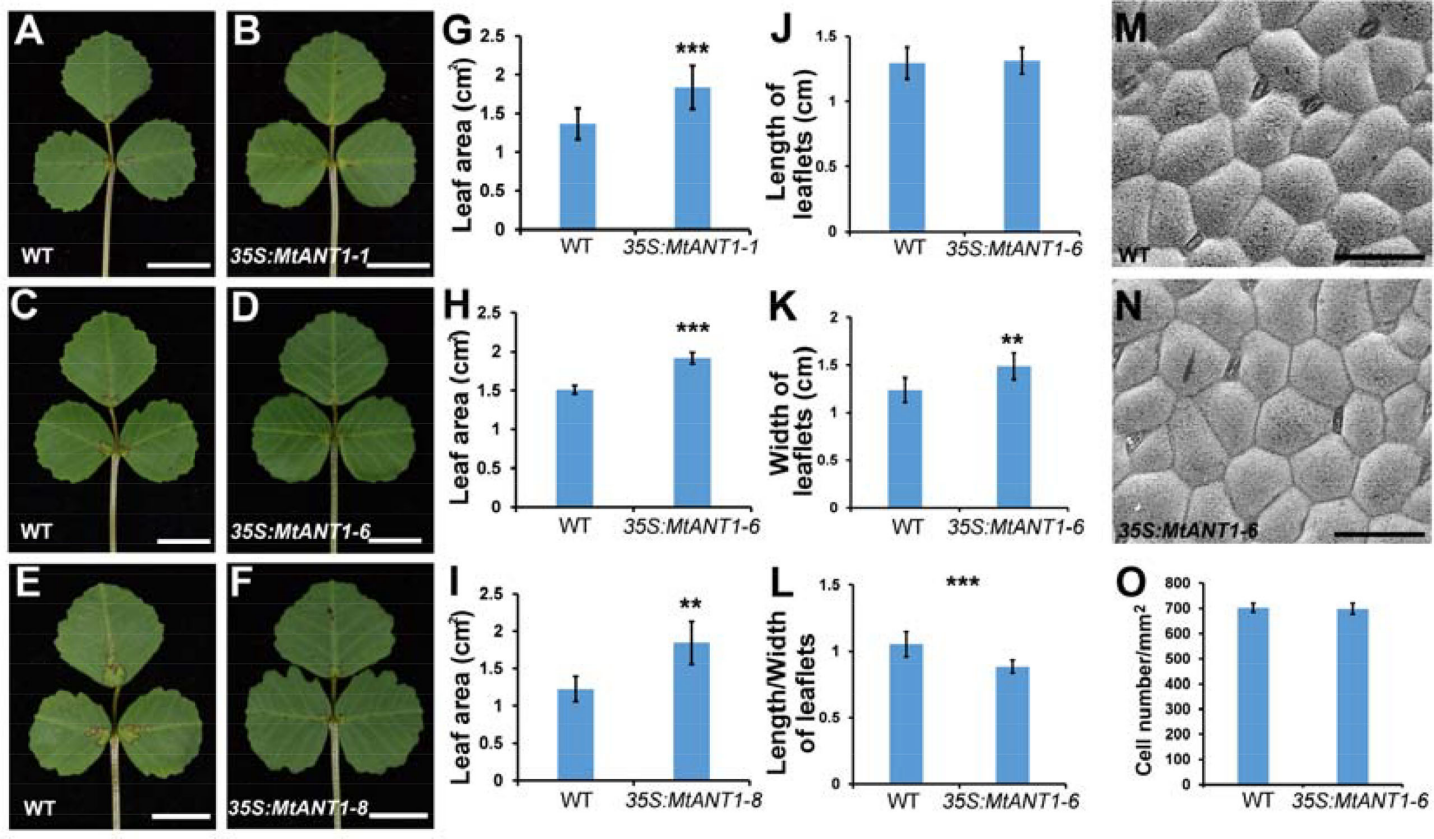
Phenotype analyses of leaves in *35S:MtANT1* plants. **(A–F)** Leaf phenotypes of wild type **(A, C, E)** and *35S:MtANT1-1*, *-6*, *-8* transgenic plants **(B, D, F)**. Bars = 1 cm. **(G–I)** Leaf area of wild type and *35S:MtANT1-1*, *-6*, *-8* plants. **(J–L)** Length **(J)**, width **(K)** and length/width ratio **(L)** of leaves in wild type and *35S:MtANT1-6* plants. **(M–N)** Scanning electron microscopy analyses of the adaxial surface of leaves in wild type **(M)** and *35S:MtANT1-6* plants **(N)** Bars = 50 μm. **(O)** Cell number per unit area of the adaxial surface of leaves in the wild type and *35S:MtANT1-6* plants. Bars represent means ± SD. ***P < 0.001, **P < 0.01.

The aforementioned findings indicated that *MtANTs* play positive roles in regulating leaf size. To determine whether increased expressions of *MtANTs* are sufficient to produce larger leaves, *MtANT1* was chosen to be overexpressed under the control of *CaMV 35S* promoter in the wild type because of its highest expression level in SAM and leaf primordia. Compared with wild type, the expression levels of *MtANT1* were increased by 93- to 669-fold in 35S*:MtANT1* transgenic plants (**Supplementary Figure S3**). Then, the leaf sizes in *35S:MtANT1* lines and wild type were compared. The *35S:MtANT1-1*, *-6*, and *-8* lines, with the highest expression levels, displayed larger leaves compared with those in wild type (**Figures 7A–F**). We measured the leaf areas of *35S:MtANT1* transgenic plants and wild type. The transgenic lines showed an increase in the area of leaves in comparison with those in wild type plants (**Figures 7G–I**). In addition, the length, width and length/width ratio of leaves of *35S:MtANT1-6* transgenic line were measured. The results showed that the length of leaves in *35S:MtANT1-6* was similar to that in the wild type, the leaves of transgenic plant were wider than wild type, and the length/width ratio of *35S:MtANT1-6* was decreased (**Figures 7J–L**). These observations suggest that ectopic expression of *MtANT1* is able to promote the leaf width and leaf size in *M. truncatula*. The epidermal cells of wild type and *35S:MtANT1-6* plants were also analyzed by SEM. The data showed that the cells size and cell number per unit area were similar between wild type and *35S:MtANT1-6* plants (**Figures 7M–O**), further demonstrating that the larger leaf area of *35S:MtANT1* transgenic plants was resulted from increased cell proliferation. Overall, *MtANTs* control leaf growth by promoting cell proliferation rather than cell expansion.

## Discussion

AINTEGUMENTA-LIKE (AIL) proteins belong to the AP2-like family, and play vital roles in plant developmental process and stress response (Horstman et al., 2014; Meng et al., 2015a; Meng et al., 2015b). AIL transcription factors have been extensively studied in different species, but their functions in the model legume, *M. truncatula*, are largely unknown. According to the phylogenetic analysis, four MtANT genes are identified. MtANT1 and MtANT3 are clustered in one clade, while MtANT2 and MtANT4 are clustered in another clade, indicating the tandem duplication followed by genomic reshuffling in *M. truncatula*. The MtAIL proteins are more closely related to *Pisum sativum* homologs, indicating that their *MtAIL* and *PsAIL* genes may diverge from a common ancestor. Compared with other AILs, most *GmAIL* genes are presented in multiple copies, suggesting that these genes are a product of whole-genome duplication events and relatively slow process of diploidization during the evolutionary process. *AIL2*/*BBM*/*PLT4*, *AIL3*/*PLT1*, *AIL4*/*PLT2* and *AIL6*/*PLT3* were reported to regulate the root stem cell niche patterning in *Arabidopsis*, and *OsPLT1*-*6* are specifically expressed in the primodium of crown root and lateral root in rice (Galinha et al., 2007; Li and Xue, 2011). Based on the phylogenetic tree analysis, we speculate that MtAIL2, 3 and 4 and their homologous proteins in the same clade among leguminous species may be involved in the regulation of root and root nodule development. Gene structure is an important indicator for gene function and classification, of which intron gain or loss is the consequence of selection pressures during evolution (Mattick, 1994). In our study, all the *MtAIL* genes evolve into the same exon-intron structures, further supporting their close evolutionary relationship. Motifs and domains are involved in various regulatory processes including interactions between proteins, transcriptional activity, and DNA binding (Liu et al., 1999). The numbers and distribution of motifs in MtAILs are different, implying that MtAIL members have some differences in function. However, two AP2 domains are highly conserved among the MtAIL proteins, suggesting that the AP2 domains are evolutionarily conserved and necessary for the correct structure of AIL proteins. Moreover, proteins sharing the unique motifs in one cluster are likely to exert similar functions (Du et al., 2012; Zhao et al., 2019b). A unique motif (motif 10) was displayed in four MtANT members. This finding raises a question on whether motif 10 is related to the leaf growth, and future characterization of the function of motif 10 will clarify this point.

*AtANT* participates in organ size control, floral organ initiation and development (Mizukami and Fischer, 2000; Horstman et al., 2014; Manchado-Rojo et al., 2014), the *ANT*-homologous genes are associated with panicle branching, panicle structure and inflorescence development in rice (Kitomi et al., 2011; Luong et al., 2021). But we observed that *MtANTs* only affect the leaf growth in *M. truncatula*. The *mtant* quadruple mutant and *MtANT1* overexpression plants didn’t exhibit other phenotypic changes, such as plant height, floral organ size and inflorescence structure. In addition, MtAIL1 is closer to MtANTs, and the developmental defects of *mtant* quadruple mutant may be masked by MtAIL1. Thus, the multiple mutations of *MtAIL1* and *MtANTs* may lead to severe developmental defects. In *Arabidopsis*, *N. tabacum*, *C. moschata*, *T. aestivum*, and *B. rapa*, ectopic expression of *ANT* enlarged the size of leaves (Mizukami and Fischer, 2000; Ding et al., 2018; Zhao et al., 2019b; Miao et al., 2021). In accordance with these reports, a similar phenotype of larger leaves was shown in *35S:MtANT1* plants. These results indicate that *ANT* genes also exert conserved function in increasing leaf area among different species. Biomass is critical for the evaluation of forage grass quality. Therefore, overexpression of *ANT* in legume forages, such as alfalfa, will be helpful to improve forage production.

Previous study showed that *ANGUSTIFOLIA3* (*AN3*) functions as a transcriptional activator of the GRF-INTERACTING FACTOR (GIF) family, and it is probably a potential target of *ANT* in promoting organ growth in *Arabidopsis* (Krizek et al., 2020). The loss-of-function mutant of *AN3* exhibits smaller leaf size, while ectopic expression of *AN3* results in larger leaves (Kim and Kende, 2004; Horiguchi et al., 2005; Lee et al., 2009). The potential regulatory relationship and phenotypes in *Arabidopsis* remind us that the paralogous gene of *AN3* in *M. truncatula* may play a similar role. In addition, *CYCD3* genes encode D-type cell cycle proteins which play key roles in the switch from cell proliferation to cell differentiation (Dewitte et al., 2003; Menges et al., 2006). The expression of *CYCD3;1* is prolonged in the leaves of *35S:ANT* plant to maintain the meristematic competence of cells during organogenesis in *Arabidopsis* (Mizukami and Fischer, 2000). Similarly, *CYCD3.2* is also the downstream target of the AIL1 transcription factor in poplar (Karlberg et al., 2011). According to these reports, it raises the possibility that *MtANTs* regulate the cell cycle genes to determine the leaf size.

The leaf development process includes three intertwined stages: leaf initiation in the SAM, primary morphogenesis, and secondary morphogenesis in which expansion and proliferation of cells occur (Poethig, 1997; Dengler and Tsukaya, 2001; Shani et al., 2009; Bar and Ori, 2014). Leaf complexity is determined during primary morphogenesis. *M. truncatula* is a compound-leafed species, whose adult leaves are trifoliate. In this study, we found the leaf complexities in both quadruple mutant and *MtANT1*-overexpressing plants were unchanged. Therefore, we propose that *MtANTs* mainly regulate the secondary morphogenesis of leaves in *M. truncatula*.

In addition, auxin plays an important role in leaf development by influencing cell proliferation and cell expansion (Tadege et al., 2011; Wang et al., 2021). In *Arabidopsis*, overexpression of the auxin-inducible gene *AUXIN-REGULATED GENE INVOLVED IN ORGAN SIZE* (*ARGOS*) generates larger leaves and prolongs the expression of *ANT*. *ARGOS* functions downstream of the auxin signaling pathway and upstream of *ANT* in the regulation of leaf size (Hu et al., 2003). So, the relationship among auxin related pathways, *MtANTs* and *MtARGOS* should be investigated in the future.

## Conclusion

In this study, we performed genome-wide analyses and identified *AIL* genes in *M. truncatula.* We characterized *MtANT* genes expression profiles in different tissues, suggesting that *MtANT* genes play important roles in the leaf morphogenesis of *M. truncatula*. Simultaneous disruption of *MtANTs* resulted in smaller leaves and overexpression of *MtANT1* led to larger leaves, demonstrating that *MtANTs* are vital for leaf size maintenance. However, they cann’t influence the leaf complexity. Further study is needed to elucidate the molecular mechanism of *MtANTs* that are involved in leaf size development.

## Data availability statement

The original contributions presented in the study are included in the article/**Supplementary Material**. Further inquiries can be directed to the corresponding author.

## Author contributions

XW, CZ and LH designed the research. XW, JJZ and JZ performed the research and analyzed the data. XW and LH wrote the paper. All authors contributed to the article and approved the submitted version.

## Funding

This study was supported by grants from the National Natural Science Foundation of China (U1906201 and 31900172) and Shandong Province (ZR2020KC018 and ZR2020QC035).

## Acknowledgments

We thank Prof. Kirankumar Mysore (Oklahoma state university), Dr. Jiangqi Wen (Oklahoma state university) for providing the *Tnt1* mutants, and Haiyan Yu from State Key Laboratory of Microbial Technology of Shandong University for help and guidance in microscope.

## Conflict of interest

The authors declare that the research was conducted in the absence of any commercial or financial relationships that could be construed as a potential conflict of interest.

## Publisher’s note

All claims expressed in this article are solely those of the authors and do not necessarily represent those of their affiliated organizations, or those of the publisher, the editors and the reviewers. Any product that may be evaluated in this article, or claim that may be made by its manufacturer, is not guaranteed or endorsed by the publisher.

## References

[B1] Aida M. Beis D. Heidstra R. Willemsen V. Blilou I. Galinha C. . (2004). The PLETHORA genes mediate patterning of the arabidopsis root stem cell niche. Cell 119 (1), 109–120. doi: 10.1016/j.cell.2004.09.018 15454085

[B2] Bar M. Ori N. (2014). Leaf development and morphogenesis. Development 141 (22), 4219–4230. doi: 10.1242/dev.106195 25371359

[B3] Boutilier K. Offringa R. Sharma V. K. Kieft H. Ouellet T. Zhang L. . (2002). Ectopic expression of BABY BOOM triggers a conversion from vegetative to embryonic growth. Plant Cell 14 (8), 1737–1749. doi: 10.1105/tpc.001941 12172019 PMC151462

[B4] Brocard-Gifford I. Lynch T. J. Garcia M. E. Malhotra B. Finkelstein R. R. (2004). The arabidopsis thaliana ABSCISIC ACID-INSENSITIVE8 encodes a novel protein mediating abscisic acid and sugar responses essential for growth. Plant Cell 16 (2), 406–421. doi: 10.1105/tpc.018077 14742875 PMC341913

[B5] Bui L. T. Pandzic D. Youngstrom C. E. Wallace S. Irish E. E. Szövényi P. . (2017). A fern AINTEGUMENTA gene mirrors BABY BOOM in promoting apogamy in ceratopteris richardii. Plant J. 90 (1), 122–132. doi: 10.1111/tpj.13479 28078730

[B6] Cheng X. Wang M. Lee H. K. Tadege M. Ratet P. Udvardi M. . (2014). An efficient reverse genetics platform in the model legume medicago truncatula. New Phytol. 201 (3), 1065–1076. doi: 10.1111/nph.12575 24206427

[B7] Chen B. Maas L. Figueiredo D. Zhong Y. Reis R. Li M. . (2022). BABY BOOM regulates early embryo and endosperm development. Proc. Natl. Acad. Sci. U.S.A. 119 (25), e2201761119. doi: 10.1073/pnas.2201761119 35709319 PMC9231476

[B8] Confalonieri M. Carelli M. Galimberti V. Macovei A. Panara F. Biggiogera M. . (2014). Seed-specific expression of AINTEGUMENTA in medicago truncatula led to the production of larger seeds and improved seed germination. Plant Mol. Biol. Rep. 32 (5), 957–970. doi: 10.1007/s11105-014-0706-4

[B9] Cosson V. Durand P. d'Erfurth I. Kondorosi A. Ratet P. (2006). Medicago truncatula transformation using leaf explants. Methods Mol. Biol. 343, 115–127. doi: 10.1385/1-59745-130-4:115 16988338

[B10] Dengler N. G. Tsukaya H. (2001). Leaf morphogenesis in dicotyledons: current issues. Int. J. Plant Sci. 162 (3), 459–464. doi: 10.1086/320145

[B11] Dewitte W. Riou-Khamlichi C. Scofield S. Healy J. M. Jacqmard A. Kilby N. J. . (2003). Altered cell cycle distribution, hyperplasia, and inhibited differentiation in arabidopsis caused by the d-type cyclin CYCD3. Plant Cell 15 (1), 79–92. doi: 10.1105/tpc.004838 12509523 PMC143452

[B12] Ding Q. Cui B. Li J. Li H. Zhang Y. Lv X. . (2018). Ectopic expression of a brassica rapa AINTEGUMENTA gene (BrANT-1) increases organ size and stomatal density in arabidopsis. Sci. Rep. 8 (1), 10528. doi: 10.1038/s41598-018-28606-4 30002453 PMC6043548

[B13] Dipp-Álvarez M. Cruz-Ramírez A. (2019). A phylogenetic study of the ANT family points to a preANT gene as the ancestor of basal and euANT transcription factors in land plants. Front. Plant Sci. 10. doi: 10.3389/fpls.2019.00017 PMC6361745 30761165

[B14] Du H. Yang S. S. Liang Z. Feng B. R. Liu L. Huang Y. B. . (2012). Genome-wide analysis of the MYB transcription factor superfamily in soybean. BMC Plant Biol. 12, 106. doi: 10.1186/1471-2229-12-106 22776508 PMC3462118

[B15] Earley K. W. Haag J. R. Pontes O. Opper K. Juehne T. Song K. . (2006). Gateway-compatible vectors for plant functional genomics and proteomics. Plant J. 45 (4), 616–629. doi: 10.1111/j.1365-313X.2005.02617.x 16441352

[B16] Galinha C. Hofhuis H. Luijten M. Willemsen V. Blilou I. Heidstra R. . (2007). PLETHORA proteins as dose-dependent master regulators of arabidopsis root development. Nature 449 (7165), 1053–1057. doi: 10.1038/nature06206 17960244

[B17] Han X. Liu K. Yuan G. He S. Cong P. Zhang C. (2022). Genome-wide identification and characterization of AINTEGUMENTA-LIKE (AIL) family genes in apple (Malus domestica borkh.). Genomics 114 (2), 110313. doi: 10.1016/j.ygeno.2022.110313 35151838

[B18] Harrop T. W. R. Mantegazza O. Luong A. M. Béthune K. Lorieux M. Jouannic S. . (2019). A set of AP2-like genes is associated with inflorescence branching and architecture in domesticated rice. J. Exp. Bot. 70 (20), 5617–5629. doi: 10.1093/jxb/erz340 31346594 PMC6812710

[B19] Hofhuis H. Laskowski M. Du Y. Prasad K. Grigg S. Pinon V. . (2013). Phyllotaxis and rhizotaxis in arabidopsis are modified by three PLETHORA transcription factors. Curr. Biol. 23 (11), 956–962. doi: 10.1016/j.cub.2013.04.048 23684976

[B20] Horiguchi G. Kim G. T. Tsukaya H. (2005). The transcription factor AtGRF5 and the transcription coactivator AN3 regulate cell proliferation in leaf primordia of arabidopsis thaliana. Plant J. 43 (1), 68–78. doi: 10.1111/j.1365-313X.2005.02429.x 15960617

[B21] Horstman A. Willemsen V. Boutilier K. Heidstra R. (2014). AINTEGUMENTA-LIKE proteins: hubs in a plethora of networks. Trends Plant Sci. 19 (3), 146–157. doi: 10.1016/j.tplants.2013.10.010 24280109

[B22] Hu Y. Xie Q. Chua N. H. (2003). The arabidopsis auxin-inducible gene ARGOS controls lateral organ size. Plant Cell 15 (9), 1951–1961. doi: 10.1105/tpc.013557 12953103 PMC181323

[B23] Karlberg A. Bako L. Bhalerao R. P. (2011). Short day-mediated cessation of growth requires the downregulation of AINTEGUMENTALIKE1 transcription factor in hybrid aspen. PloS Genet. 7 (11), e1002361. doi: 10.1371/journal.pgen.1002361 22072988 PMC3207903

[B24] Kim J. H. Kende H. (2004). A transcriptional coactivator, AtGIF1, is involved in regulating leaf growth and morphology in arabidopsis. Proc. Natl. Acad. Sci. U.S.A. 101 (36), 13374–13379. doi: 10.1073/pnas.0405450101 15326298 PMC516574

[B25] Kim S. Soltis P. S. Wall K. Soltis D. E. (2006). Phylogeny and domain evolution in the APETALA2-like gene family. Mol. Biol. Evol. 23 (1), 107–120. doi: 10.1093/molbev/msj014 16151182

[B26] Kitomi Y. Ito H. Hobo T. Aya K. Kitano H. Inukai Y. (2011). The auxin responsive AP2/ERF transcription factor CROWN ROOTLESS5 is involved in crown root initiation in rice through the induction of OsRR1, a type-a response regulator of cytokinin signaling. Plant J. 67 (3), 472–484. doi: 10.1111/j.1365-313X.2011.04610.x 21481033

[B27] Klucher K. M. Chow H. Reiser L. Fischer R. L. (1996). The AINTEGUMENTA gene of arabidopsis required for ovule and female gametophyte development is related to the floral homeotic gene APETALA2. Plant Cell 8 (2), 137–153. doi: 10.1105/tpc.8.2.137 8742706 PMC161087

[B28] Krizek B. (2009). AINTEGUMENTA and AINTEGUMENTA-LIKE6 act redundantly to regulate arabidopsis floral growth and patterning. Plant Physiol. 150 (4), 1916–1929. doi: 10.1104/pp.109.141119 19542297 PMC2719149

[B29] Krizek B. A. (2015). AINTEGUMENTA-LIKE genes have partly overlapping functions with AINTEGUMENTA but make distinct contributions to arabidopsis thaliana flower development. J. Exp. Bot. 66 (15), 4537–4549. doi: 10.1093/jxb/erv224 25956884 PMC4507765

[B30] Krizek B. A. Bantle A. T. Heflin J. M. Han H. Freese N. H. Loraine A. E. (2021). AINTEGUMENTA and AINTEGUMENTA-LIKE6 directly regulate floral homeotic, growth, and vascular development genes in young arabidopsis flowers. J. Exp. Bot. 72 (15), 5478–5493. doi: 10.1093/jxb/erab223 34013313 PMC8318262

[B31] Krizek B. A. Blakley I. C. Ho Y. Y. Freese N. Loraine A. E. (2020). The arabidopsis transcription factor AINTEGUMENTA orchestrates patterning genes and auxin signaling in the establishment of floral growth and form. Plant J. 103 (2), 752–768. doi: 10.1111/tpj.14769 32279407 PMC7369219

[B32] Kuluev B. Avalbaev A. Nurgaleeva E. Knyazev A. Nikonorov Y. Chemeris A. (2015). Role of AINTEGUMENTA-like gene NtANTL in the regulation of tobacco organ growth. J. Plant Physiol. 189, 11–23. doi: 10.1016/j.jplph.2015.08.009 26479044

[B33] Kumar S. Nei M. Dudley J. Tamura K. (2008). MEGA: a biologist-centric software for evolutionary analysis of DNA and protein sequences. Brief Bioinform. 9 (4), 299–306. doi: 10.1093/bib/bbn017 18417537 PMC2562624

[B34] Larkin M. A. Blackshields G. Brown N. P. Chenna R. McGettigan P. A. McWilliam H. . (2007). Clustal W and clustal X version 2.0. Bioinformatics 23 (21), 2947–2948. doi: 10.1093/bioinformatics/btm404 17846036

[B35] Lee B. H. Ko J. H. Lee S. Lee Y. Pak J. H. Kim J. H. (2009). The arabidopsis GRF-INTERACTING FACTOR gene family performs an overlapping function in determining organ size as well as multiple developmental properties. Plant Physiol. 151 (2), 655–668. doi: 10.1104/pp.109.141838 19648231 PMC2754652

[B36] Licausi F. Ohme-Takagi M. Perata P. (2013). APETALA2/Ethylene responsive factor (AP2/ERF) transcription factors: mediators of stress responses and developmental programs. New Phytol. 199 (3), 639–649. doi: 10.1111/nph.12291 24010138

[B37] Liu W. Y. Lin H. H. Yu C. P. Chang C. K. Chen H. J. Lin J. J. . (2020). Maize ANT1 modulates vascular development, chloroplast development, photosynthesis, and plant growth. Proc. Natl. Acad. Sci. U.S.A. 117 (35), 21747–21756. doi: 10.1073/pnas.2012245117 32817425 PMC7474692

[B38] Liu L. White M. J. MacRae T. H. (1999). Transcription factors and their genes in higher plants functional domains, evolution and regulation. Eur. J. Biochem. 262 (2), 247–257. doi: 10.1046/j.1432-1327.1999.00349.x 10336605

[B39] Li P. Xue H. (2011). Structural characterization and expression pattern analysis of the rice PLT gene family. Acta Biochim. Biophys. Sin. (Shanghai) 43 (9), 688–697. doi: 10.1093/abbs/gmr068 21807632

[B40] Luong A. M. Adam H. Gauron C. Affortit P. Ntakirutimana F. Khong N. G. . (2021). Functional diversification of euANT/PLT genes in oryza sativa panicle architecture determination. Front. Plant Sci. 12. doi: 10.3389/fpls.2021.692955 PMC8302143 34305984

[B41] Manchado-Rojo M. Weiss J. Egea-Cortines M. (2014). Validation of aintegumenta as a gene to modify floral size in ornamental plants. Plant Biotechnol. J. 12 (8), 1053–1065. doi: 10.1111/pbi.12212 24985495

[B42] Mattick J. S. (1994). Introns: evolution and function. Curr. Opin. Genet. Dev. 4 (6), 823–831. doi: 10.1016/0959-437x(94)90066-3 7888751

[B43] Menges M. Samland A. K. Planchais S. Murray J. A. (2006). The d-type cyclin CYCD3;1 is limiting for the G1-to-S-phase transition in arabidopsis. Plant Cell 18 (4), 893–906. doi: 10.1105/tpc.105.039636 16517759 PMC1425856

[B44] Meng L. S. Wang Y. B. Yao S. Q. Liu A. (2015a). Arabidopsis AINTEGUMENTA mediates salt tolerance by trans-repressing SCABP8. J. Cell Sci. 128 (15), 2919–2927. doi: 10.1242/jcs.172072 26054800

[B45] Meng L. S. Wang Z. B. Yao S. Q. Liu A. (2015b). The ARF2-ANT-COR15A gene cascade regulates ABA-signaling-mediated resistance of large seeds to drought in arabidopsis. J. Cell Sci. 128 (21), 3922–3932. doi: 10.1242/jcs.171207 26395398

[B46] Miao L. Li S. Z. Shi A. K. Li Y. S. He C. X. Yan Y. . (2021). Genome-wide analysis of the AINTEGUMENTA-like (AIL) transcription factor gene family in pumpkin (Cucurbita moschata duch.) and CmoANT1.2 response in graft union healing. Plant Physiol. Biochem. 162, 706–715. doi: 10.1016/j.plaphy.2021.03.036 33799182

[B47] Mizukami Y. Fischer R. L. (2000). Plant organ size control: AINTEGUMENTA regulates growth and cell numbers during organogenesis. Proc. Natl. Acad. Sci. U.S.A. 97 (2), 942–947. doi: 10.1073/pnas.97.2.942 10639184 PMC15435

[B48] Mizumoto K. Hatano H. Hirabayashi C. Murai K. Takumi S. (2009). Altered expression of wheat AINTEGUMENTA homolog, WANT-1, in pistil and pistil-like transformed stamen of an alloplasmic line with aegilops crassa cytoplasm. Dev. Genes Evol. 219 (4), 175–187. doi: 10.1007/s00427-009-0275-y 19255779

[B49] Mudunkothge J. S. Krizek B. A. (2012). Three arabidopsis AIL/PLT genes act in combination to regulate shoot apical meristem function. Plant J. 71 (1), 108–121. doi: 10.1111/j.1365-313X.2012.04975.x 22380923

[B50] Nole-Wilson S. Krizek B. A. (2006). AINTEGUMENTA contributes to organ polarity and regulates growth of lateral organs in combination with YABBY genes. Plant Physiol. 141 (3), 977–987. doi: 10.1104/pp.106.076604 16714408 PMC1489906

[B51] Nole-Wilson S. Tranby T. L. Krizek B. A. (2005). AINTEGUMENTA-like (AIL) genes are expressed in young tissues and may specify meristematic or division-competent states. Plant Mol. Biol. 57 (5), 613–628. doi: 10.1007/s11103-005-0955-6 15988559

[B52] Pinon V. Prasad K. Grigg S. P. Sanchez-Perez G. F. Scheres B. (2013). Local auxin biosynthesis regulation by PLETHORA transcription factors controls phyllotaxis in arabidopsis. Proc. Natl. Acad. Sci. U.S.A. 110 (3), 1107–1112. doi: 10.1073/pnas.1213497110 23277580 PMC3549086

[B53] Poethig R. S. (1997). Leaf morphogenesis in flowering plants. Plant Cell 9 (7), 1077–1087. doi: 10.1105/tpc.9.7.1077 9254931 PMC156981

[B54] Prasad K. Grigg S. P. Barkoulas M. Yadav R. K. Sanchez-Perez G. F. Pinon V. . (2011). Arabidopsis PLETHORA transcription factors control phyllotaxis. Curr. Biol. 21 (13), 1123–1128. doi: 10.1016/j.cub.2011.05.009 21700457

[B55] Radović J. Sokolović D. Marković J. (2009). Alfalfa-most important perennial forage legume in animal husbandry. Biotechnol. Anim. Husbandry 25 (5-6-1), 465–475. doi: 10.2298/BAH0906465R

[B56] Rigal A. Yordanov Y. S. Perrone I. Karlberg A. Tisserant E. Bellini C. . (2012). The AINTEGUMENTA LIKE1 homeotic transcription factor PtAIL1 controls the formation of adventitious root primordia in poplar. Plant Physiol. 160 (4), 1996–2006. doi: 10.1104/pp.112.204453 23077242 PMC3510126

[B57] Shani E. Burko Y. Ben-Yaakov L. Berger Y. Amsellem Z. Goldshmidt A. . (2009). Stage-specific regulation of solanum lycopersicum leaf maturation by class 1 KNOTTED1-LIKE HOMEOBOX proteins. Plant Cell 21 (10), 3078–3092. doi: 10.1105/tpc.109.068148 19820191 PMC2782295

[B58] Shen S. Sun F. Zhu M. Chen S. Guan M. Chen R. . (2020). Genome-wide identification AINTEGUMENTA-like (AIL) genes in brassica species and expression patterns during reproductive development in. PloS One 15 (6), e0234411. doi: 10.1371/journal.pone.0234411 32511257 PMC7279594

[B59] Tadege M. Lin H. Bedair M. Berbel A. Wen J. Rojas C. M. . (2011). STENOFOLIA regulates blade outgrowth and leaf vascular patterning in medicago truncatula and nicotiana sylvestris. Plant Cell 23 (6), 2125–2142. doi: 10.1105/tpc.111.085340 21719692 PMC3160033

[B60] Tadege M. Wen J. He J. Tu H. Kwak Y. Eschstruth A. . (2008). Large-Scale insertional mutagenesis using the Tnt1 retrotransposon in the model legume medicago truncatula. Plant J. 54 (2), 335–347. doi: 10.1111/j.1365-313X.2008.03418.x 18208518

[B61] Wang H. Kong F. Zhou C. (2021). From genes to networks: The genetic control of leaf development. J. Integr. Plant Biol. 63 (7), 1181–1196. doi: 10.1111/jipb.13084 33615731

[B62] Wang H. Lu Z. Xu Y. Kong L. Shi J. Liu Y. . (2019). Genome-wide characterization of SPL family in medicago truncatula reveals the novel roles of miR156/SPL module in spiky pod development. BMC Genomics 20 (1), 552. doi: 10.1186/s12864-019-5937-1 31277566 PMC6612136

[B63] Wang H. Wang H. Liu R. Xu Y. Lu Z. Zhou C. (2018). Genome-wide identification of TCP family transcription factors in medicago truncatula reveals significant roles of miR319-targeted TCPs in nodule development. Front. Plant Sci. 9. doi: 10.3389/fpls.2018.00774 PMC6004737 29942322

[B64] Yamaguchi N. Jeong C. W. Nole-Wilson S. Krizek B. A. Wagner D. (2016). AINTEGUMENTA and AINTEGUMENTA-LIKE6/PLETHORA3 induce LEAFY expression in response to auxin to promote the onset of flower formation in arabidopsis. Plant Physiol. 170 (1), 283–293. doi: 10.1104/pp.15.00969 26537561 PMC4704571

[B65] Yang T. Liu R. Luo Y. Hu S. Wang D. Wang C. . (2022). Improved pea reference genome and pan-genome highlight genomic features and evolutionary characteristics. Nat. Genet. 54 (10), 1553–1563. doi: 10.1038/s41588-022-01172-2 36138232 PMC9534762

[B66] Zhang J. Wang X. Han L. Zhang J. Xie Y. Li J. . (2022). The formation of stipule requires the coordinated actions of the legume orthologs of arabidopsis BLADE-ON-PETIOLE and LEAFY. New Phytol. 236(4), 1512–1528. doi: 10.1111/nph.18445 36031740

[B67] Zhao Y. Liu R. Xu Y. Wang M. Zhang J. Bai M. . (2019a). AGLF provides c-function in floral organ identity through transcriptional regulation of AGAMOUS in medicago truncatula. Proc. Natl. Acad. Sci. U.S.A. 116 (11), 5176–5181. doi: 10.1073/pnas.1820468116 30782811 PMC6421450

[B68] Zhao Y. Ma R. Xu D. Bi H. Xia Z. Peng H. (2019bb). Genome-wide identification and analysis of the AP2 transcription factor gene family in wheat (Triticum aestivum l.). Front. Plant Sci. 10. doi: 10.3389/fpls.2019.01286 PMC6797823 31681381

[B69] Zhou C. Han L. Hou C. Metelli A. Qi L. Tadege M. . (2011). Developmental analysis of a medicago truncatula smooth leaf margin1 mutant reveals context-dependent effects on compound leaf development. Plant Cell 23 (6), 2106–2124. doi: 10.1105/tpc.111.085464 21693694 PMC3160044

[B70] Zhu H. Choi H. K. Cook D. R. Shoemaker R. C. (2005). Bridging model and crop legumes through comparative genomics. Plant Physiol. 137 (4), 1189–1196. doi: 10.1104/pp.104.058891 15824281 PMC1088312

