# Supplementary material for: Genome-wide characterization of AINTEGUMENTA-LIKE family in Medicago truncatula reveals the significant roles of AINTEGUMENTAs in leaf growth
Source: Front Plant Sci. 2022 Nov 3;13:1050462. doi: 10.3389/fpls.2022.1050462 (PMC9669440; doi:10.3389/fpls.2022.1050462)
Supplement: Supplementary file 1 [file DataSheet_1.doc]

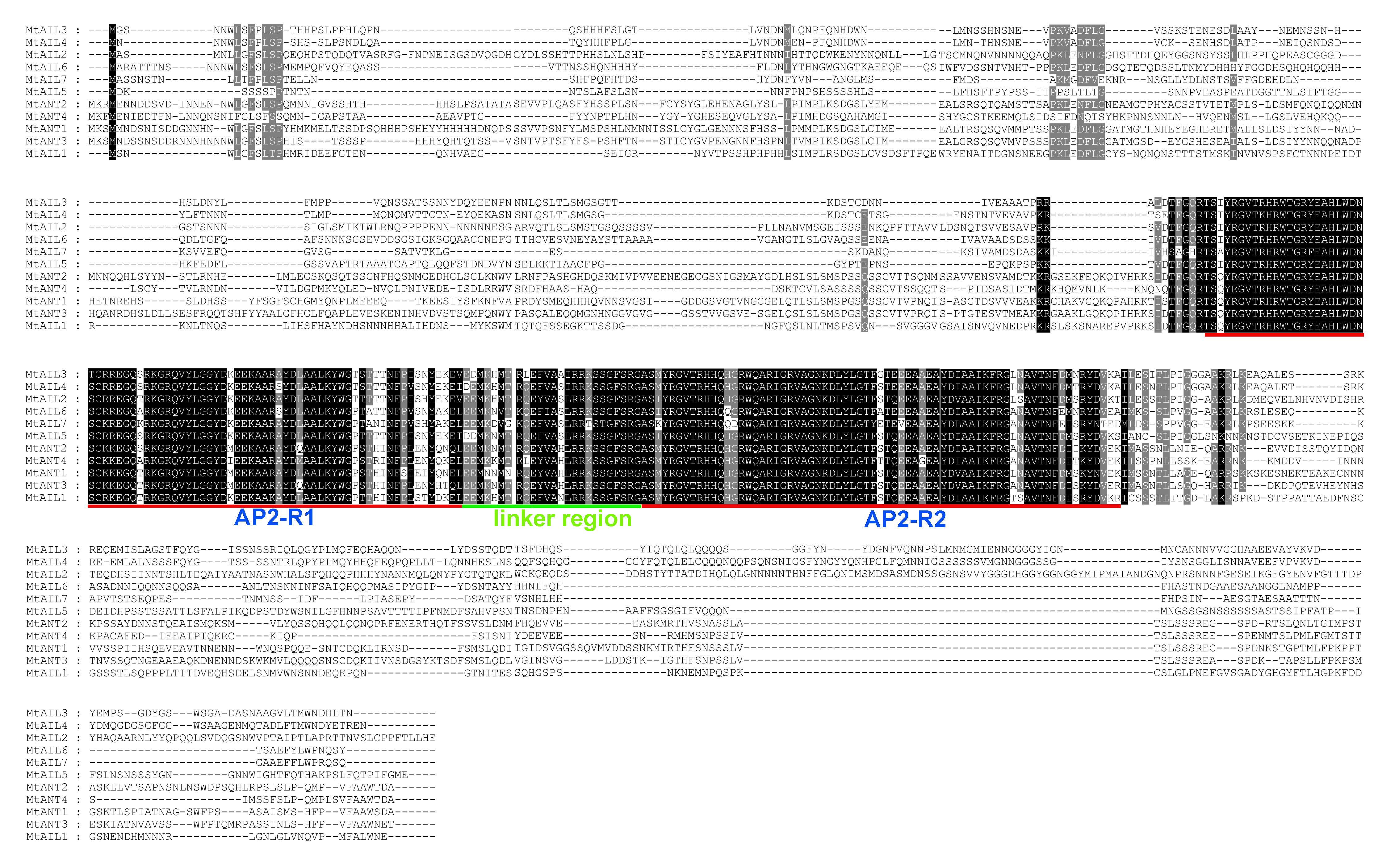


**Supplementary Figure 1.** Alignment of the AIL protein sequences in *M. truncatula*. Protein sequence alignment was performed using Clustal X2, two AP2 domains and the linker region were shown.


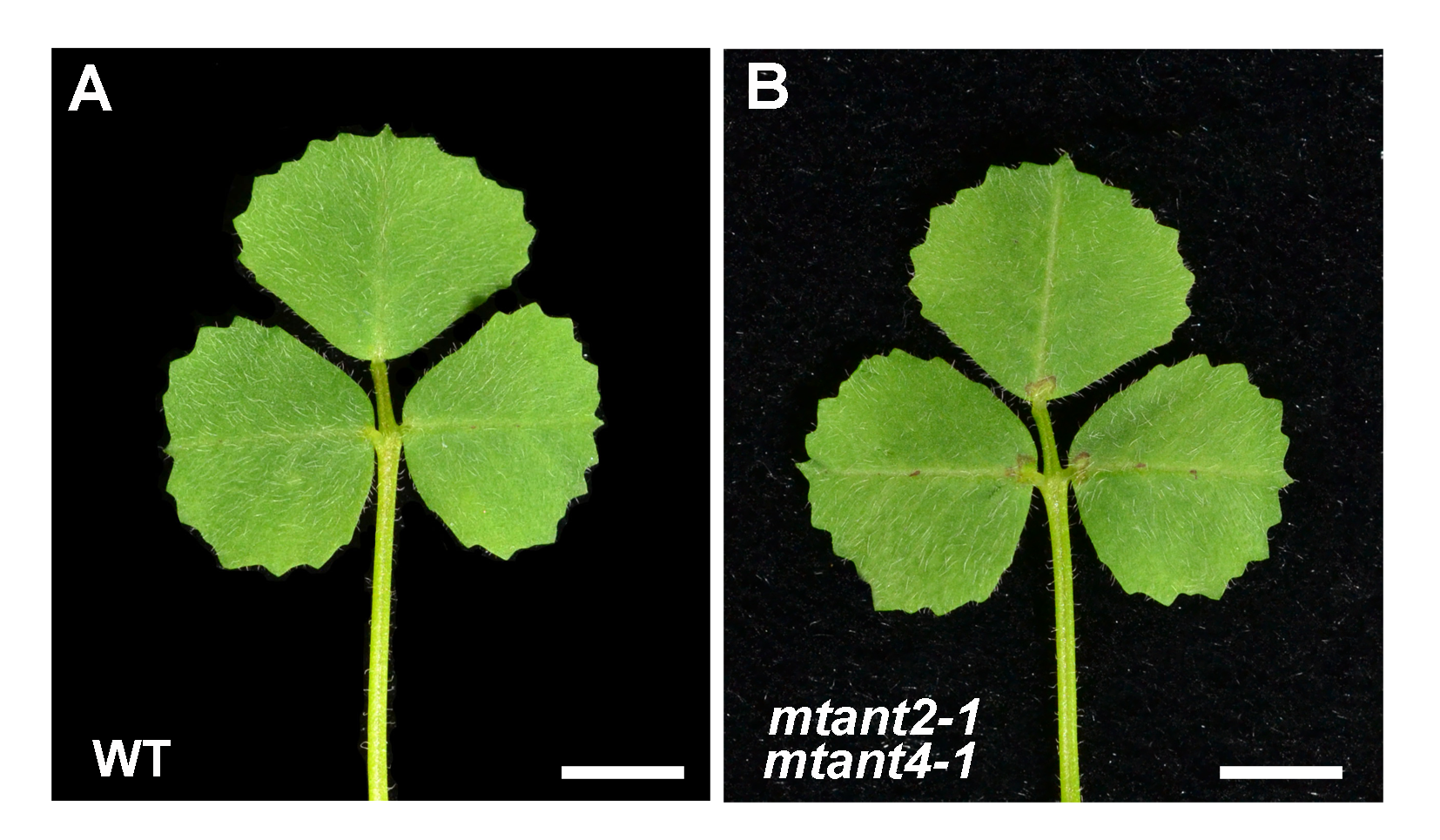


**Supplementary Figure 2.** The leaf phenotypes of wild type **(A)** and *mtant2-1 mtant4-1* double mutant **(B)**.


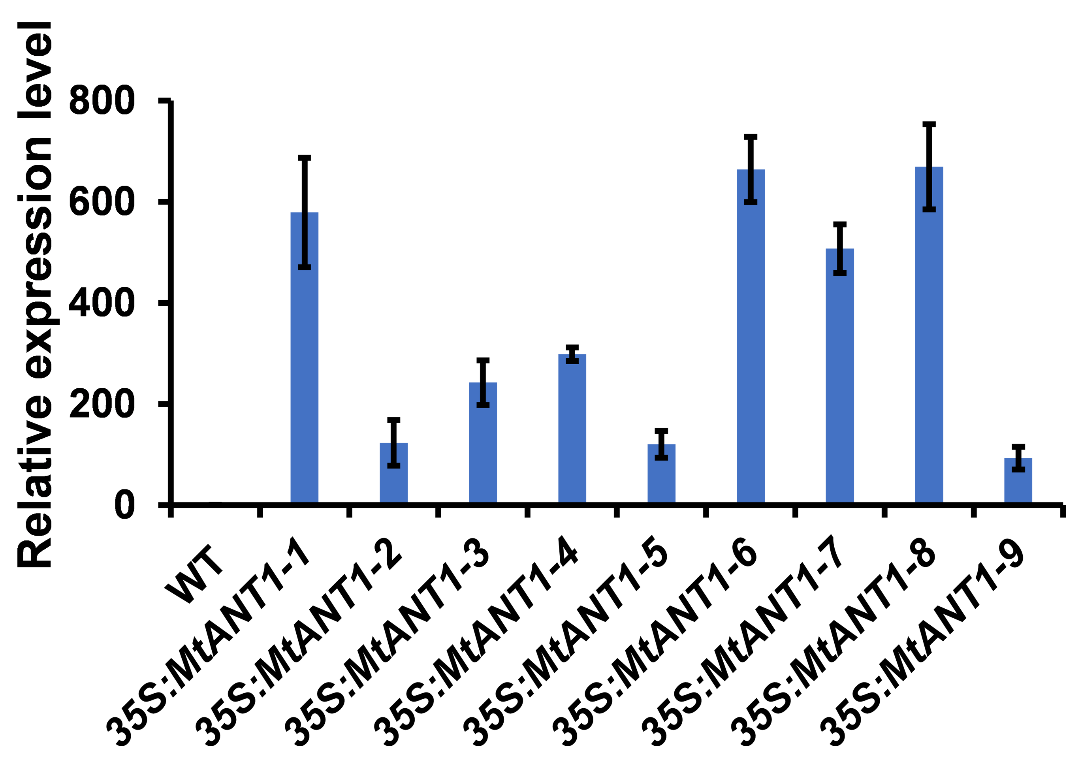


**Supplementary Figure 3.** The expression levels of *MtANT1* in the leaves of wild type and *35S:MtANT1* transgenic lines determined by qRT-PCR.

**Supplementary** Table 1 Primers used in this study

| **Primer** | **Sequence** | **Application** |
| --- | --- | --- |
| MtANT1-QF | CACCTTGCTTGCTGGTGAAC | For qRT-PCR analysis of *MtANT1* |
| MtANT1-QR | TGACTCTTGTTGTGGGGACTG |
| MtANT2-QF | CCAACATGGAAGATGGCAAGC | For qRT-PCR analysis of *MtANT2* |
| MtANT2-QR | TGGTCACTGCATTAGCTCCTC |
| MtANT3-QF | GTGGATTCTCAAGGGGTGCT | For qRT-PCR analysis of *MtANT3* |
| MtANT3-QR | TCCAATCCTAGCTTGCCACC |
| MtANT4-QF | GAGGGCCAAGCTAGAAAAGGA | For qRT-PCR analysis of *MtANT4* |
| MtANT4-QR | CGAGTGGAGGGTCCCCAATA |
| MtANT1-CF | CACC ATGAAGTCCATGATGAATGATAGTAAC | For cloning of the *MtANT1* full length CDS |
| MtANT1-CR | AGCATCACTCCAAGCCGC |
| MtANT1-prob-F1 | GGAAGGGCAAACAAGGAAAGG | For cloning of the *MtANT1* as a probe for *in situ* hybridization |
| MtANT1-prob-R1 | GGTCACAGCTTCAACTTCTTGAC |
| MtANT2-prob-F1 | GGTTAGGTTTTTCACTCTCTCCTC | For cloning of the *MtANT2* as a probe for *in situ* hybridization |
| MtANT2-prob-R1 | CTTCACCCATGTTTGATTGATGG |
| MtANT3-prob-F1 | ATGAAGTCCATGAATGACAGTAG | For cloning of the *MtANT3* as a probe for *in situ* hybridization |
| MtANT3-prob-R1 | GAAGGTCAAGGGAATGGTCTC |
| MtANT1-RT-F1 | GTGGTTTTTCAAGGGGTGCTTC | For RT-PCR analysis of *MtANT1* |
| MtANT1-RT-R1 | GGTCACAGCTTCAACTTCTTGAC |
| MtANT2-RT-F1 | GGTTAGGTTTTTCACTCTCTCCTC | For RT-PCR analysis of *MtANT2* |
| MtANT2-RT-R1 | CTTCACCCATGTTTGATTGATGG |
| MtANT3-RT-F1 | ATGAAGTCCATGAATGACAGTAG | For RT-PCR analysis of *MtANT3* |
| MtANT3-RT-R1 | GAAGGTCAAGGGAATGGTCTC |
| MtANT4-RT-F1 | CACCAGAAGCAACAGCTCTC | For RT-PCR analysis of *MtANT4* |
| MtANT4-RT-R1 | GATGTTCTTTGCCCAAAGGTTTG |
